# Non-Coding RNA Regulatory Network in Ischemic Stroke

**DOI:** 10.3389/fneur.2022.820858

**Published:** 2022-03-03

**Authors:** Zongyan Cai, Shuo Li, Tianci Yu, Jiahui Deng, Xinran Li, Jiaxin Jin

**Affiliations:** ^1^The Second Clinical Medical College, Lanzhou University, Lanzhou, China; ^2^Department of Orthopedics, The Second Hospital of Lanzhou University, Lanzhou, China

**Keywords:** non-coding RNA, ischemic stroke, regulating network, lncRNA, circRNA

## Abstract

Stroke is a worldwide public health problem that has caused a substantial economic burden to families and society. Despite recent major advances, there is still a need for more timely, effective diagnosis and treatment methods for acute ischemic stroke. Non-coding RNAs (ncRNAs), which widely exist in the human body, do not encode proteins. Instead, these mediate various cellular processes as functional regulatory molecules from the RNA level. Each ncRNA node in organisms is not isolated but constitutes a complex regulatory network, regulating multiple molecular targets and triggering specific physiological or pathological reactions, leading to different outcomes. Abundant studies have proclaimed the impact of ncRNAs in ischemic stroke, which may enlighten new inspirations for diagnosing and treating ischemic stroke. This paper outlines the current understanding of the ncRNA regulatory network and reviews the recent evidence for the contribution of ncRNAs in the experimental ischemic stroke model.

## Introduction

Stroke is a severe life-threatening acute cerebrovascular disease, which is mainly divided into ischemic stroke (IS) and hemorrhagic stroke. Furthermore, a proportion of hemorrhagic strokes can be secondary to IS. This is called the hemorrhagic transformation of brain infarction and is a complication of IS (1). Ischemic stroke, which accounts for the majority of strokes, is caused by a sharp decrease in cerebral blood flow, which will further cause hypoxia and tissue damage. The subsequent mechanism involves many pathological processes, such as energy failure, acidosis, calcium overload, excitotoxicity, mitochondrial damage, oxidative stress, inflammatory reaction, etc. (2). IS can ultimately cause cell necrosis or apoptosis, leading to a neurological deficit of the corresponding cerebral area and resulting in adverse outcomes. In terms of diagnosis, due to the rapid onset and few precursor symptoms, the diagnosis of IS is often based on the clinical and imaging manifestations after the disease onset. Hence, there is still a lack of sensitive early biomarkers. The key principle of IS treatment is “time is brain," which means that early recanalization of blood supply to the ischemic area is very important to improve the prognosis. At present, the standard treatment of IS is intravenous injection of recombinant tissue plasminogen activator (rtPA) and mechanical treatment to remove the clot to achieve vascular recanalization (3). However, the indications, complications, and possible ischemia-reperfusion injury of thrombolytic therapy limit its universal application in clinics. Therefore, it is essential to explore the molecular mechanisms underlying IS's pathogenesis to improve the diagnosis and treatment of the disease.

Non-coding RNA (ncRNA) widely exists in organisms. It does not encode proteins but serves as functional RNA which mainly regulates post-transcriptional gene expression. In the narrow sense, non-coding RNAs mainly include microRNA (miRNA), circular RNA (circRNA), and long non-coding RNA (lncRNA). miRNA is a small molecule with a length of about 21 nucleotides. It can cause silencing or degradation of target messenger RNA (mRNA) by binding to the 3′-untranslated region (3′-UTR) of mRNA, regulating gene expression at the post-transcriptional level. miRNAs are expressed in all human tissues and are tissue- and time-specific. The relationship between miRNA and mRNA is not one-to-one. miRNAs can be divided into hundreds of families. Different members of the same miRNA family can target multiple genes and jointly regulate specific physiological processes. The same mRNA molecule can be regulated by multiple upstream miRNAs as well, and it can subsequently participate in multiple downstream pathways. Multiple nodes are interconnected to form a very complex and precise regulation network. Studies have shown that miRNA is involved in regulating almost all cell biological processes, and its expression changes are related to a variety of pathological processes (4). It has become a critical factor in the post-transcriptional regulation of gene expression. lncRNA is a non-coding RNA with a length >200 nucleotides. It possesses a wide range of functions and complex mechanisms to regulate the body's growth and development. For example, post-transcriptional regulation is one of the functions that can be achieved in various ways. lncRNA can directly interact with proteins, bind specific transcription factors as molecular bait to prevent it from binding with DNA, and regulate miRNA expression as a miRNA sponge or enhancer (5). circRNA is a circular single-stranded RNA molecule, which can be covalently closed by reverse splicing of mRNA precursor transcripts in eukaryotic cells. Unlike linear molecules, their 5′ and 3′ ends are directly connected to form a circular closed structure without free ends. At present, it has been found that circRNA can regulate transcription, splicing, and chromatin interactions. Moreover, it could directly bind to proteins and enter the cytoplasm to serve as endogenous competitive RNA (ceRNA) to prevent the binding of miRNAs to target mRNAs (6). The expression level of circRNAs in cells is generally low, and its expression is also tissue-specific. Significant enrichment of circRNAs can be observed in the brain, and they can participate in the disease progression (7). However, hitherto, the research on its function is not sufficient.

Non-coding RNAs (ncRNAs) are abundant in the brain. The level of ncRNAs in the brain can change to varying degrees at the time of the onset of neurovascular disease, forming a characteristic ncRNA expression profile. The change of ncRNA expression level after IS is the response of the body toward injury factors. Its follow-up effect has advantages and disadvantages for the progress and prognosis of the disease. Current studies have identified the role of ncRNAs' imbalance in the onset and development of IS and proposed the potential of ncRNAs to be novel biomarkers applied in clinical diagnosis and treatment. However, due to the complexity and multi-targeting of the ncRNA regulation mechanism, the relationship between different ncRNA nodes and between ncRNAs and downstream molecules remain unexplored. So, there are significant limitations in the clinical transformation of basic research. In this review, we innovatively classified the miRNAs involved in the stroke process into protective and damaging miRNAs according to their post-disease expression levels and relationships with prognosis to analyze their functions and effects in the disease progression or deterioration. Meanwhile, we preliminarily explored the potential molecular mechanism and regulatory network of ncRNAs in IS, forming various lncRNA/circRNA-miRNA-downstream molecular axes and constructing a primary regulatory network of ncRNAs, discussed ncRNA's significance as a biomarker and therapeutic target, and prospected the research prospect of ncRNA.

## Alterations in Ncrna Expression Following Ischemic Stroke

At present, many ncRNAs with abnormal expression in the occurrence and development of IS have been found, and many studies have explored the regulatory relationship of ncRNAs under the guidance of miRNAs. So far, miRNA has been proved to be involved in neuroprotection and repair, brain injury and cell death, changes in neuronal excitability, glial scar formation, and so forth (8). Therefore, we will describe the ncRNA expression profile of IS based on the expression change of miRNAs.

The expression level of miRNAs changes rapidly after ischemia, presenting diverse expression patterns in different periods. Jeyaseelan et al. analyzed the expression level of miRNAs in brain tissue and blood in 24–48 h after middle cerebral artery occlusion reperfusion and confirmed the abnormal expression of miRNAs of IS and firstly reported the role and molecular mechanism of miRNAs in the middle cerebral artery occlusion-related diseases (9). This part will classify the miRNAs related to the IS into protective and damaging types based on the expression alteration of miRNAs detected after the onset of IS, the effects of alteration, and the impact of the use of antagonists or agonists on the prognosis. The presence of protective miRNAs means that the alteration of miRNAs expression level after IS is advantageous to the prognosis, and reversing its expression has an opposite effect (Table 1). On the other hand, damaging miRNAs refer to miRNAs which expression level alteration after IS is unfavorable to prognosis, and reversing its expression level can reduce pathological damage (Table 2). In the existing research data, the expression of some miRNAs changes over time, making it difficult to define the benefits or harm of their function. Hence, they are not within the scope of discussion.

**Table 1:** Protective microRNA (miRNA) and its upstream and downstream molecules and main functions.

**miRNAs**	**Up/** **down**	**Target molecule/pathway**	**Affected** **pathological processes**	**Influence on pathological processes**	**Upstream** **molecules**	**Up/down**
miR-26a (10)	Up	PI3K/AKT MAPK/ERK VEGF	cell proliferation Angiogenesis	Alleviated	-	-
miR-128 (11)	Up	MAPK	-	Alleviated	-	-
miR-146a (12)	Up	IRAK1	oligodendrogenesis	Alleviated	-	-
miR-124 (13)	Up	PI3K/AKT/mTOR caspase-3 Bcl-2 Bcl-xl	Apoptosis Autophagy	Alleviated	-	-
miR-199a (14)	Down	SIRT1 MAPK	cell proliferation	Alleviated	lncRNA-SNHG12	Up
miR-130a-5p (15)	Down	VEGF-A	Angiogenesis	Alleviated	lncRNA-MEG8	Up

**Table 2:** Damaging miRNA and its upstream and downstream molecules and main functions.

**miRNAs**	**Up/** **down**	**Target** **molecule/pathway**	**Affected** **pathological processes**	**Influence on pathological processes**	**Upstream** **molecules**	**Up/** **down**
miR-497 (16)	Up	Bcl-2/Bcl-w	Apoptosis	Aggravated	-	-
miR-503 (17)	Up	PI3K/Akt/eNOS Bcl-2, caspsase-3	Apoptosis Oxidative stress	Aggravated	-	-
miR-191 (18)	Up	VEZF1	Angiogenesis	Aggravated	-	-
miR-210 (19)	Up	TNF-α/IL-1β/IL-6	Inflammation	Aggravated	-	-
miR-449c-5p (20)	Up	STAT6	Neuroinflammation	Aggravated	lncRNA-SHNG4	Down
miR-186-5p (21)	Up	CTRP3	inflammation of microglia/macrophage Oxidative stress	Aggravated	lncRNA-OIP5-AS1	Down
miR-125b-5p (22)	Up	GDF11	Apoptosis	Aggravated	circRNA-UCK2	Down
miR-582-3p (23)	Up	NOS3	Neuroinflammation Apoptosis oxidative stress	Aggravated	lncRNA-ZFAS1	Down
miR-145 (24, 25)	Down	AQP4	apoptosis	Aggravated	lncRNA-TUG1 lncRNA-MALAT1	Up
miR-195 (26)	Down	Bcl-2/JNK/KLF5	Neuroinflammation Apoptosis	Aggravated	-	-
miR-let-7c-5p (27)	Down	Caspase-3	Apoptosis	Aggravated	-	-
miR-24 (28)	Down	Caspase-3	Apoptosis	Aggravated	-	-
miR-92b (29)	Down	NOX4	BBB damage	Aggravated	FOXO1	Up
miR-375 (30)	Down	PDE4D	Apoptosis neuroinflammation	Aggravated	lncRNA-MALAT1	Up
miR-30a (31)	Down	Beclin1	Autophagy	Aggravated	lncRNA-MALAT1	Up
miR-181b (32)	Down	12/15-LOX/HSPA5/UCHL1	Apoptosis	Aggravated	lncRNA-MEG3	Up
miR-424-5p (33)	Down	Sema3A	apoptosis	Aggravated	lncRNA-MEG3	Up
miR-147 (34)	Down	SOX2/NF-kB/ Wnt/β-catenin	Apoptosis	Aggravated	lncRNA-MEG3	Up
miR-21 (35)	Down	PTEN/PI3K/AKT/PDCD4	Apoptosis	Aggravated	lncRNA-Gas5 lncRNA-MEG3	Up
miR-181c-5p (36–38)	Down	HMGB1/BIM/BMF	Apoptosis	Aggravated	lncRNA-SNHG6 lncRNA-MALAT1 lncRNA-SNHG14	Up
miR-136-5p (39)	Down	ROCK1	Neuroinflammation	Aggravated	lncRNA-SNHG14	Up
miR-30b-5p (40)	Down	Atg5/Beciln1	Autophagy	Aggravated	lncRNA-SNHG14	Up
miR-199b (41)	Down	MAPK/ERK/Egr1/AQP4	macrophages apoptosis cell proliferation	Aggravated	lncRNA-SNHG14	Up
miR-183-5p	Down	FOXO1/PI3K/Akt	apoptosis	Aggravated	lncRNA-SNHG15	Up
miR-19a-3p	Down	PTEN/PI3K/AKT	Apoptosis oxidative stress	Aggravated	lncRNA-H19	Up
miR-153-3p (42)	Down	FOXO3	-	Aggravated	lncRNA-KCNQ1OT1	Up
miR-142 (43)	Down	Beclin1/TIPARP	Autophagy	Aggravated	circRNA-HECTD1	Up
miR-335-3p (44)	Down	TIPARP	apoptosis	Aggravated	circRNA-TLK1	Up
miR-133b (45)	Down	TRAF1 NF-κB	Apoptosis	Aggravated	circRNA-HECTD1	Up
miR-204-5p (46)	Down	HMGB1	Angiogenesis Inflammation	Aggravated	lncRNA-MIAT	Up
miR-130a-3p (47)	Down	DAPK1	Apoptosis	Aggravated	lncRNA-H19	Up
miR-24-3p (48)	Down	Nrp1/NF-κB	Apoptosis	Aggravated	lncRNA-THRIL	Up
miR-214 (49)	Down	PI3K/AKT VEGF	Apoptosis angiogenesis	Aggravated	lncRNA-NEAT1	Up
miR-26a-5p (50)	Down	DAPK1	apoptosis	Aggravated	lncRNA-AK038897	Up
miR-335 (51)	Down	ROCK1/AKT/GSK-3β	apoptosis	Aggravated	lncRNA-Gas5	Up
miR-455-5p (52)	Down	PTEN	Apoptosis oxidative stress	Aggravated	lncRNA-Gas5	Up
miR-19a (53)	Down	Id2	apoptosis	Aggravated	lncRNA-H19	Up
miR-200a-3p (54)	Down	NLPR3	Neuroinflammation	Aggravated	lncRNA-TUG1	Up
miR-874-3p (55)	Down	IL1B	Apoptosis neuroinflammation	Aggravated	lncRNA-MIAT	Up

Neuronal apoptosis and necrosis are the most severe and common pathological changes of IS. This process is mediated by a variety of mechanisms, including but not limited to excitotoxicity, oxidative stress, inflammation, ion imbalance and secondary edema, transcription factor failure, endoplasmic reticulum stress, mitochondrial dysfunction, and abnormal methylation (56). The neurons' regeneration potentiality of the central nervous system is low (57). Once dead, the neuron will be phagocytosed by microglia and replaced by a glial scar, leaving neurological defects. Yin et al. found that miRNA-497 increased significantly within 24 h after middle cerebral artery embolization and verified it with the glucose oxygen deprivation model (OGD) (16). The results showed that the high expression of miR-497 would aggravate neuronal damage. miR-497 directly binds to the predicted 3′-UTR target sites of bcl-2/-w genes and downregulates its expression level. Knockout of miR-497 can increase the level of Bcl-2/-w protein in the ischemic area, reducing the area of cerebral infarction and improving neurological function. Aquaporin 4 (AQP4), a protein on the cell membrane, can control the water molecules moving in and out of cells and help water molecules pass through the blood-brain barrier (BBB). The high abnormal expression of AQP4 can aggravate the cell damage caused by IS, leading to a poor prognosis. miR-145 has been proved to regulate AQP4 (24, 25). After IS, the expression level of miR-145 decreased, but AQP4 increased, showing a higher level of apoptosis. Accordingly, the use of miR-145 siRNA (small interfering RNA, which can lead to the silencing of target miRNAs complementary to it) reduced the damage and produced a protective effect.

Cysteine proteases (caspases) are a highly conserved family of cysteine proteases. There are many family members. Among them, hitherto 11 family members have been found. The imbalance of caspase activation plays an essential role in inflammation and tumorigenesis. At present, many studies have shown that caspase is abnormally expressed in IS and regulated by multiple miRNAs (58). For example, miR-let-tc-3p, miR-195, miR-24, miR-233, miR-503, etc., have been proved to be related to this process (17, 26–28, 59). miR-130a was upregulated in brain tissue of MCAO rats (60) and X-Linked Inhibitor Of Apoptosis (XIAP) was identified as its target molecule. XIAP is an inhibitor of caspase 3/7/9 and belongs to the inhibitor of the apoptosis protein family. It can be observed that the level of XIAP was upregulated when the expression of miR-130a was inhibited. Correspondingly, apoptosis reduced and angiogenesis increased to improve the neural function of rats. Other studies have shown that the high expression of miR-130a in serum is related to the adverse consequences of acute intracerebral hemorrhage and perihematomal edema, which can increase BBB permeability and aggravate inflammatory reaction during cerebral ischemia (61). Interestingly, the expression of miR-130a in neurons of MCAO rats is low, while upregulating miR-130a can inhibit phosphatase and tensin homolog (PTEN) expression and enhance PI3K/AKT pathway activity from preventing ischemia-reperfusion injury. The phenomenon suggests that the expression of miRNA is different inside and outside the cell. Some miRNAs can be induced to a high expression level and then released out of the cells in certain forms, resulting in the rise of miRNA expression in the tissue (2). The effect of miR-191 is similar to miR-130a (18). It was overexpressed in acute ischemic stroke brain tissue and preferentially expressed in endothelial cells. Its effects lie in anti-angiogenesis and inhibit endothelial cell proliferation and migration. miR-191 can inhibit the expression of Vascular Endothelial Zinc Finger 1 (VEZF1), thus inhibiting angiogenesis and promoting ischemia-reperfusion injury.

The inflammatory response is an adaptive response secondary to infection and tissue injury. Typically, the BBB limits the circulating immune cells and molecules to enter the brain. When the brain injury occurs, the BBB is damaged and demonstrates an increased permeability. Peripheral immune cells can pass through the BBB together with the innate immune cells and inflammatory mediators of brain tissue, which triggers the post-injury inflammatory response. Brain inflammation after IS is characterized by microglial activation and circulating inflammatory cell infiltration (62). Hypoxia due to ischemia is the main pathogenic factor of IS. Hypoxia is accompanied by the rapid increase of inflammatory mediators. The inflammatory response can cause irreversible damage to neurons in the ischemic area and lead to neurological dysfunction. The expression of miR-92b decreases in brain microvascular endothelial cells (BMECs) after IS, which is suppressed by upstream molecular transcription factor Forkhead Box O1 (FOXO1) that is induced to be highly expressed by IS. The promotion of the low expression of miR-92b is associated with lower BBB permeability, which was originally induced to elevate by the OGD. Intracerebroventricular injection of miR-92b adenovirus vector can improve the survival rate of BMECs and inhibit the expression of downstream nicotinamide adenine dinucleotide phosphate (NADPH) oxidase 4 (NOX4) to improve the stability of the BBB (29).

Some miRNA expressions can be induced by hypoxia. These miRNAs, such as miR-199a, miR-107, miR-210, and so on, are called hypoxia-induced miRNAs (hypoximiRs) (63, 64). As master hypoxamiR, miR-210 plays an essential role in fine-tuning the adaptive response of cells toward hypoxia and can be strongly induced by hypoxia (65). Many studies have proved that miR-210 is induced to be highly expressed in MCAO, and the application of miR-210-locking nucleotide (LNA, a kind of miRNA inhibitor) can reduce TNF-α level immediately after IS and completely block IL-1β, and the expression of pro-inflammatory factor IL-6 is blocked 12 h after IS, but the expression of anti-inflammatory factor such as TGF-β and IL-10 is insusceptible. At the same time, it can also inhibit the expression of chemokines CCL2 and CCL3. These two chemokines are excreted by activated microglia and injured endothelial cells after IS to recruit inflammatory cells to pass through the BBB and trigger ischemic inflammation, suggesting that miR-210-LNA plays a neuroprotective role by antagonizing pro-inflammatory factors and inhibiting inflammatory infiltration (19). However, some studies have found that exosomes containing miR-210 can induce angiogenesis after injection into the cerebral ventricle of MCAO mice (66). It can directly target cytokine Suppressor Of Cytokine Signaling 1 (SOCS1) and increase the expression of STAT3 and VEGF-C to promote angiogenesis around ischemic foci and the aggregation of neural precursor cells (NPCs), which is good for IS prognosis (67).

## Ncrna Regulatory Network in Ischemic Stroke

**Figure 1:**
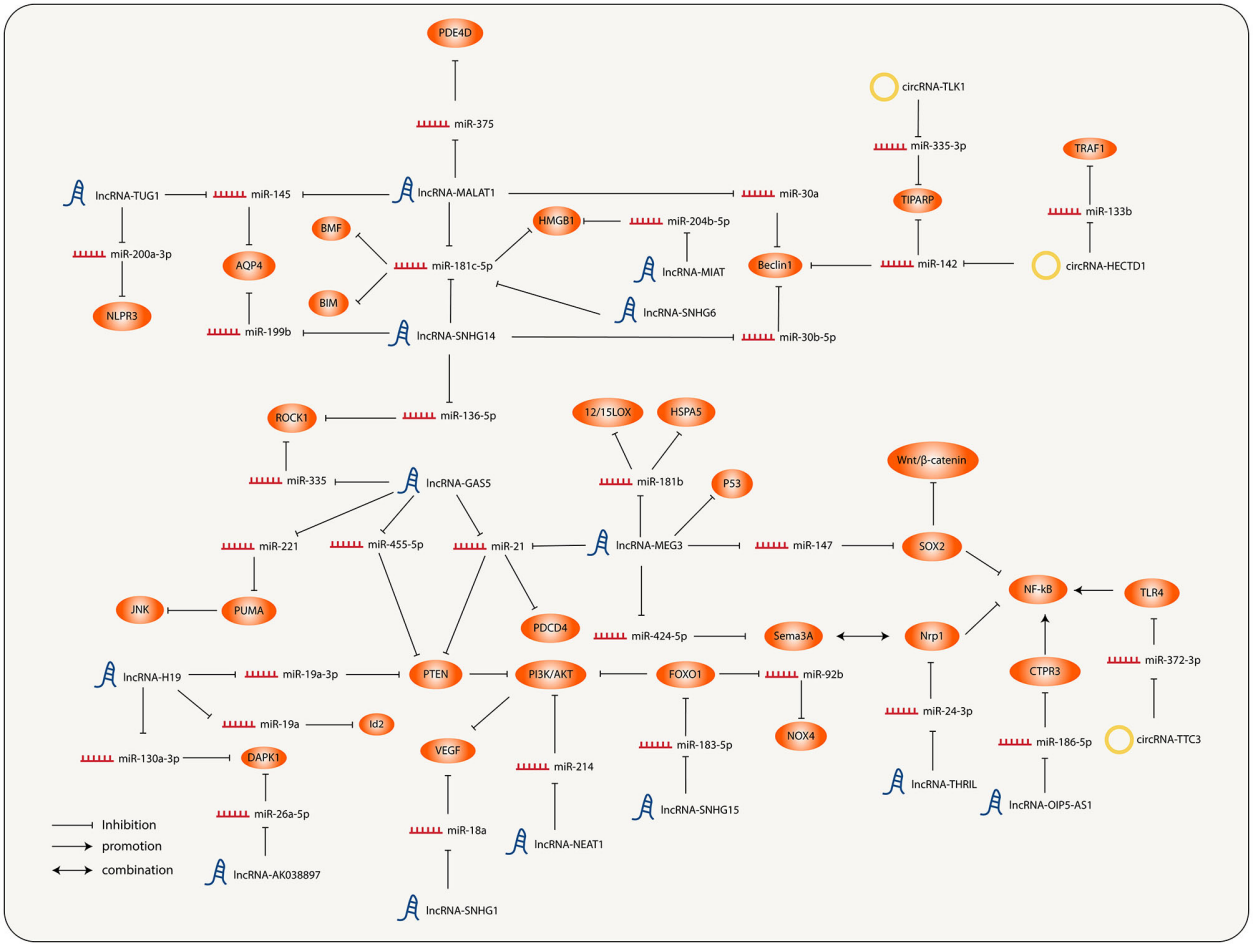
Interconnection and regulation of the different molecular nodes involved in the progression of ischemic stroke (IS).

It has been previously described that both lncRNAs and circRNAs act as miRNA sponges. Therefore, a large number of lncRNAs and circRNAs together with their regulated miRNAs and miRNAs targeting molecules constitute the lncRNA/circRNA-miRNA-mRNA axis, and interconnection of each axis ultimately forms a complicated ncRNA regulatory network of IS (Figure 1).

### Long Non-Coding RNAs and MicroRNAs

A large number of studies have demonstrated that the pathology of the central nervous system is concerned with the abnormal expression of lncRNA. A variety of lncRNAs have been found to be involved in the onset and development of IS for several years. In addition, lncRNA can also be utilized as a potential new biomarker for disease diagnosis, treatment, and prognosis.

In recent years, lncRNA-MALAT1 has been observed to express abnormally in IS and participate in some pathological processes. The expression of MALAT1, the function of which refers to inflammatory and apoptosis pathways, was significantly upregulated in patients' microglia and neurons. miR-375 is one of the target molecules of MALAT1. The decrease causes high expression of Phosphodiesterase 4D (PDE4D) through the miR-375/PDE4D axis, promoting inflammation and apoptosis (30). MALAT1 can also target miR-181c-5p and upregulate the expression of High Mobility Group Box 1 (HMGB1), a pro-inflammatory factor (36), aggravating the degree of inflammation. Jin et al. found that the expression levels of MALAT1 and AQP4 were consistent (68). Relevant studies have shown that MALAT1 can increase the level of AQP4 and cause a larger infarct area by inhibiting miR-145 (24). Autophagy is a necessary evolutionarily conserved process of substance turnover within eukaryotic cells, also reflected in neural injury. In IS, autophagy can be activated under excitotoxic conditions, and autophagy activated by neurons under cerebral ischemia is adverse (69). MALAT1 can upregulate the expression of Beclin1 by inhibiting miR-30a (31). High levels of Beclin-1 will increase autophagy and deteriorate neuronal damage.

To sum up, the high expression of lncRNA-MALAT1 is related to more severe inflammation and autophagy, and it also spawns larger infarct size, which is unfavorable to the prognosis of IS. However, some studies have found that high expression of MALAT1 can improve the prognosis in IS. Zhang et al. found that the decrease of MALAT1 expression would increase ischemia-induced endothelial cell loss, apoptosis, and inflammation (70). Silencing MALAT1 provoked the expression of pro-apoptotic factor Bcl-2 interacting mediator of cell death (BIM), pro-inflammatory factor Monocyte Chemoattractant Protein-1 (MCP-1), IL-6, and E-selectin, significantly increasing caspase-3 activity and inducing BMECs' death.

As with MALAT1, the expression level of lncRNA-MEG3 also increased after IS. It can directly target miR-181b (32). The inhibitory effect of miR-181b on 12/15-lipoxygenase (LOX) was relieved, which exacerbated hypoxia-induced neuronal apoptosis. MEG3 can also target miR-424-5p, resulting in increased expression of downstream semaphorin 3A (Sema3A) (33), thereby activating the MAPK signal pathway associated with Sema3A and hampering the axon growth. Similarly, other studies have proved that Sema3A inhibitors can activate the Akt pathway and phosphorylate Glycogen synthase kinase-3β (GSK-3β). The phosphorylation of GSK-3β suppresses astrocyte activation and promotes axon regeneration after ischemia (71). GSK-3 is a highly conserved serine-threonine kinase that was initially identified as phosphorylation and inactivator of glycogen synthase. It has two isoforms, α and β. GSK-3β is confirmed to participate in energy metabolism, inflammation, endoplasmic reticulum stress, mitochondrial dysfunction, apoptosis, etc., making it possible to become a new therapeutic target of IS (72). At the same time, Sema3A can also competitively bind Neuropilin 1 (NRP-1) and antagonize VEGF to prevent angiogenesis. In addition, MEG3 could target miR-21, increasing the expression of Programmed Cell Death 4 (PDCD4), a pro-apoptotic protein, and hindering the neuron-protective effect of miR-21. Inhibiting MEG3's expression level can boost nerve growth and reduce nerve injury led by ischemia-reperfusion (73). Furthermore, MEG3 was found to act on the Notch pathway and Wnt/β-catenin pathway, causing inhibition of angiogenesis and simultaneously increasing neuronal apoptosis (74). This finding was supplemented by Han et al. who argued that miR-147 and downstream molecule SRY-Box Transcription Factor 2 (SOX2) were involved in the effect of MEG3 on the regulation of Wnt/β-Catenin pathway and NF- κB pathway (34), and that the high level of SOX2 will activate the above pathways and cause more severe hypoxia injury.

Studies have confirmed that lncRNA-GAS5 negatively regulates multiple miRNAs. High expression of GAS5 in the brain after IS can block the cell cycle and increase apoptosis (75). Firstly, the GAS5 expression level was observed to be consistent with PTEN expression. Relevant studies pointed out that the effect could be mediated by miR-21 and miR-455-5p (35, 52). High levels of PTEN act as negative regulators to dephosphorylate AKT and reduce activation of which, inhibiting PI3K/AKT pathway, further affects neuronal survival, leading to cell apoptosis, oxidative damage, and mitochondrial damage, accelerating the progression of ischemic brain injury. In addition, Zhou et al. predicted and verified the accurate binding site between GAS5 and miR-221 (76). The rise of GAS5 level leads to the upregulation of p53 upregulated modulator of apoptosis (PUMA), further resulting in dephosphorylation and inactivation of histone H2AX through the JNK pathway and causing more severe cell apoptosis. On the contrary, the expression of miR-221 increased after the knockout of GAS5. Consequently, the expression of PUMA decreased, and the apoptosis decreased correspondingly.

Small nucleolar RNA host genes (SNHGs) are a group of lncRNAs that have been proved to be oncogenes of many cancers. Recent studies have found that abnormal expression of SNHG1/4/6/12/14/15 exists in the process of IS. SNHG1 expressed significantly high in the MCAO mouse model and OGD-cultured microvascular endothelial cells, which function as a protective role. Knockdown of SNHG1 was correlated with higher caspase-3 activity and more severe apoptosis (77). Moreover, SNHG1 also acts as a ceRNA for miR-18a, resulting in elevated downstream HIF-1α expression and pro-proliferation of vascular endothelial cells *via* the HIF-1α/VEGF axis. It was also shown that SNHG4 has a regulatory effect on the severity of microglia inflammation (20) by comparing the content of lncRNA in serum and cerebrospinal fluid samples of MCAO model mice, patients with acute cerebral infarction, and ordinary people. Zhang et al. found that the expression of SNHG4 in microglia of patients and MCAO mice was significantly lower than the normal level. When increasing the expression level of SNHG4 and STAT6, the target molecule of miR-449c-5p was upregulated, which could inhibit the inflammatory response after cerebral ischemia-reperfusion injury.

The SNHG6 expression level was significantly upregulated after IS (37). It can act as the ceRNA of miR-181c-5p, relieving the transcriptional inhibition of miRNA on the downstream target gene BIM and causing more severe apoptosis. Knockout of SNHG6 can improve cell survival, suppress caspase-3 activity, and alleviate nerve injury. SNHG12 level was also notably raised after ischemia or reperfusion injury treatment (14, 78). Overexpressed SNHG12 targets miR-199a, promotes the expression of NAD-Dependent Protein Deacetylase Sirtuin-1(SIRT1), and further activates AMPK pathway and boosts cell proliferation, while inhibition of SNHG12 can reverse this effect. ncRNA-SNHG14 is a lncRNA that is widely upregulated in a variety of diseases (38). At present, many pathways that involve SNHG14 in IS have been found. The expression of SNHG14 increases in the brain of ischemia-reperfusion mice, which combines with miR-136-5p and stimulates the expression of downstream molecule ROCK1 and promotes inflammatory response (39). miR-181c-5p is also one of the downstream molecules of SNHG14 (38). BCL2 modifying factor (BMF) is a member of the Bcl-2 homologous domain 3 (BH3) protein family. As a pro-apoptotic protein, it was found to be negatively regulated by miR-181c-5p. SNHG14 positively regulates BMF expression and aggravates neuronal injury. The knockdown of SNHG14 can significantly promote OGD-induced neuronal proliferation, inhibit apoptosis, and play an anti-inflammatory role. Previously, miR-181c-5p has also been proved to be regulated by lncRNA MALAT1 (46), which suggests that miRNA itself is regulated in many aspects.

It is worth noting that some studies have pointed out that the expression of miR-181c-5p is upregulated in myocardial cells after ischemia-reperfusion and can aggravate the level of apoptosis and inflammation (79). This is opposite to the effect of miR-181c-5p in the brain, suggesting that the regulatory effects of ncRNAs vary in different cell types. Sun et al. argued that SNHG14 can target miR-30b-5p to promote the expression of Autophagy Related 5 (ATG5) and Beclin1 to activate autophagy and enhance brain inflammation level (40). They also found that transcription factor SP1 directly interacted with the promoter of SNHG14 and thus upregulated the expression of SNHG14. Meanwhile, SP1 itself was also regulated by P38/MAPK pathway, indicating that lncRNA itself was also regulated by upstream pathways and molecules. Otherwise, studies have demonstrated that it is through miR-199b/AQP4 axis that SNHG14 can promote inflammation and oxidative stress (41, 80). SNHG15 is highly expressed in MCAO rats according to the research of Wen et al., which can act as the ceRNA of miR-183-5p, upregulating the expression of FOXO1 and cyclin-dependent kinase inhibitor 1B (P27^Kip1^) and resulting in cell cycle block and apoptosis.

The expression of lncRNA-H19 was upregulated in MCAO mice (81). It mediates the expression of PTEN through the downstream molecule miR-19a-3p. The upregulation of the PTEN level causes the inactivation of the PI3K/AKT pathway and aggravates oxidative stress and apoptosis. Knockout of H19 can alleviate the damage. The expression of lncRNA-KCNQ1OT1 was significantly increased in neurons of MCAO mice. Studies showed that its role in promoting neuronal injury was achieved by regulating the expression of FOXO3 as a ceRNA of miR-153-3p (42). OPA-interacting protein 5 antisense RNA1 (OIP5-AS1) is a recently discovered long non-coding RNA (21). It was downregulated in microglia of ischemic brain tissue, resulting in low expression of C1q/TNF-Related Protein 3 (CTRP3) by targeting miR-186-5p, thereby activating Nrf2 and NF-κB pathway, significantly promoting inflammation and oxidative stress response, and showing greater infarct size. At the same time, it has been proved that there is cross-talk between Nrf2 and NF-κB pathways. The interaction between Nrf2 and NF-κB pathways can make cells regulate their response more finely to stressors and improve their adaptability to environmental changes. Upregulation of OIP5-AS1 can increase the expression of CTPR3 and reduce the damage response induced by microglia and macrophages.

It should be noted that lncRNA acts in a wide range of ways *in vivo*. In addition to inhibiting the expression of miRNA through the molecular sponge, it can also directly interact with proteins and participate in pathophysiological processes. As Yan et al. (56) found the DBD^270−281^ region of P53 is responsible for the direct association between MEG3 and P53. MEG3 can positively regulate P53 and activate its transcriptional activity, mediating neuronal death after IS.

### Circular RNA and MicroRNAs

**Figure 2:**
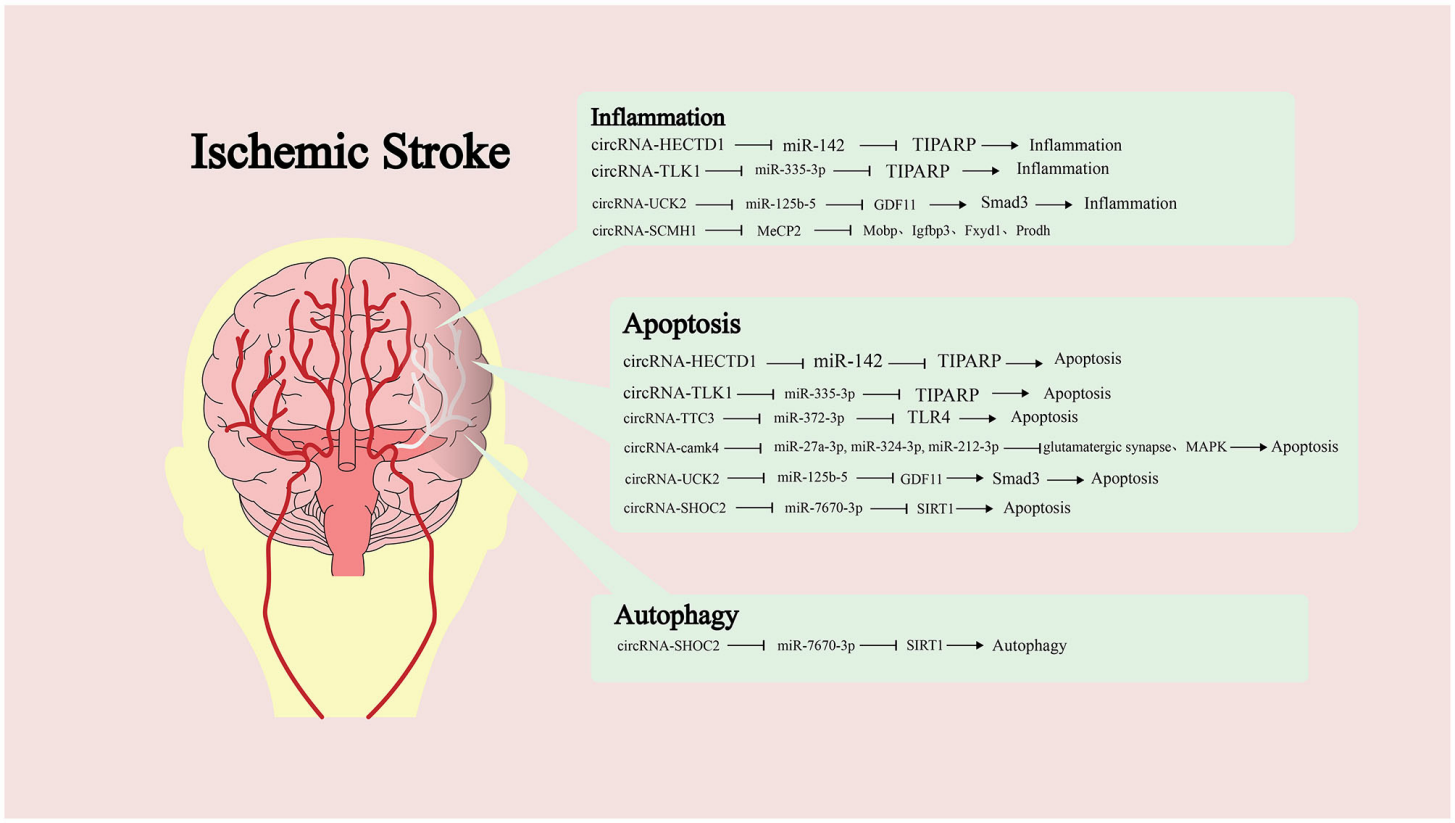
Targeting of downstream microRNAs (miRNAs) by different circular RNAs (circRNAs) and the molecules or pathways are ultimately affected, along with the impact on pathological processes.

Many studies have shown that the imbalance of circRNA expression is one of the characteristics of IS (82). However, the research on the role of circRNA in IS is not sufficient. Dong et al. compared the circRNA expression profiles of monocytes from ordinary people and patients with IS (83). Bioinformatics analysis showed that abnormally expressed circRNA was involved in multiple pathophysiological processes, such as inflammation and immune processes. Therefore, it is of positive significance to study the role of circRNA in IS (Figure 2).

Han et al. found that the expression of circRNA-HECTD1 was significantly increased in ischemic brain tissue of MCAO mice and plasma of patients with acute ischemic stroke (AIS) (43). It can be used as ceRNA of miR-142 to inhibit the translation inhibition of TCDD inducible poly (ADP ribose) polymerase (TIPARP) by miR-142. The highly expressed TIPARP can increase the activation of astrocytes and promote inflammation and apoptosis. Knockout of HECTD1 can significantly reduce infarct size and regulate astrocyte autophagy and activation. Similarly, the expression of circRNA-TLK1 increased in ischemic brain tissue (44). By inhibiting miR-335-3p, it leads to the decrease of TIPARP expression, which aggravates the neuronal injury. Neural stem cells (NSCs) are a kind of cells with self-renewal ability and multi-differentiation potential. After brain injury, endogenous static NSCs are activated. These then participate in the process of brain repair and play an essential role in IS (84). Yang et al. explored the effects of circRNA-TTC3 on ischemia-reperfusion injury and NSCs (85). TTC3 expression was significantly increased in hypoxic astrocytes, and its target was miR-372-3p. TTC3 depletion could lead to high expression of Toll-like receptor 4 (TLR4), inhibiting the activation of the OGD-induced apoptosis pathway and promoting the proliferation of NSCs. Zhang et al. found that highly expressed circRNA-camk4 after IS can significantly increase the cell mortality after ischemia-reperfusion in MCAO rats (82). In addition, they predicted and confirmed that miR-27a-3p, miR-324-3p, and miR-212-3p were the target molecules of camk4. The regulation of miRNA by camk4 involves the glutamatergic synaptic pathway, MAPK pathway, and apoptosis pathway.

The expression level of circRNA-UCK2 significantly decreased in the brain tissue of the ischemia-reperfusion mouse model (22). In contrast, increasing its expression can significantly reduce the size of the infarct area and improve the neurological deficit. It can inhibit the expression of miR-125b-5p and increase the expression of growth differentiation factor-11 (GDF11) and bind TGF-β Receptor to activate the downstream Smad3 signaling pathway, resulting in anti-apoptotic and anti-inflammatory effects. GDF11 is a member of the activin subfamily and TGF-β superfamily that binds two types of TGF- β receptors (TβRI and TβRII) to form an active signal transduction complex. TβRII activates TβRI kinase activity by phosphorylating TβRI before transmitting signals intracellularly *via* phosphorylating transcription factor Smad by TβRI. Smads mainly accumulate in the nucleus and regulate the expression of target genes by combining DNA and other transcription mechanisms. circRNA can also participate in the regulation of autophagy (86). circRNA-SHOC2 is highly expressed in ischemic-preconditioned astrocyte-derived exosomes (IPAS-EXOs). SHOC2 can target miR-7670-3p, increase the expression of downstream molecule SIRT1, and inhibit neuronal apoptosis. At the same time, SHOC2 can also reduce neuronal damage by regulating autophagy.

Circular RNA (circRNA) can directly function with targeted protein molecules. Yang et al. screened circRNA-SCMH1 from the plasma of patients with IS and found that its expression level significantly decreased after IS (87). After treating brain tissue with SCMH1 analogs, the functional recovery after IS was improved while enhancing neuronal plasticity and inhibiting glial cell activation and peripheral immune cell infiltration. The downstream target search results show that it could directly bind to the transcription factor Methyl-CpG Binding Protein 2 (MeCP2) and attenuate the transcriptional repression of MeCP2 on the target genes Myelin Associated Oligodendrocyte BasicProtein (Mobp), Insulin-Like Growth Factor Binding Protein 3 (Igfbp3), FXYD Domain Containing Ion Transport Regulator 1 (Fxyd1), and Proline Dehydrogenase 1(Prodh), all of which play an important role in maintaining brain function.

## Clinical Significance

A large number of studies have confirmed that ncRNAs can be utilized as new biomarkers and therapeutic targets. More and more pieces of evidence accumulated over the years show that miRNA can exist in tissues and various biological fluids, such as blood, urine, saliva, and so on (88). miRNA circulates in a highly stable acellular form in peripheral blood and can be detected in plasma or serum (64). Many studies have shown that nerve cells can somehow secrete miRNA after IS, providing a theoretical basis for ncRNA expression profile as a biomarker in IS. Zuo et al. identified the differentially expressed circRNA in IS patients' blood (89) before isolating platelets, lymphocytes, and granulocytes from the blood to identify the source of circRNA. It was identified that the expression of circFUNDC1, circPDS5B, and circCDC14A in plasma was higher than in the standard control group and positively correlated with infarct volume. PDS5B and CDC14A may come from lymphocytes and granulocytes. When three circRNAs were utilized in combination to detect IS, they showed high sensitivity and specificity. However, the limitation is that there is no sufficient research evidence based on lncRNA and circRNA targeted therapy after IS, but these ncRNAs involved in the pathological process of IS have the potential to become therapeutic targets.

As mentioned earlier, miRNA expression is disordered after IS, and abnormal expression of some miRNAs is related to poor prognosis. To improve the prognosis of patients, ncRNA antagonists or analogs can be applied to reverse the expression of ncRNAs at the pathological level to play a protective role. Liu et al. injected a miR-21/miR-24 inhibitor into the cerebral ventricle of MCAO rats and found that it can significantly reduce the apoptosis of hippocampal and cortical neurons (90). Early intraventricular injection of miR-124 has been shown to significantly improve neuronal survival and microglia M2-like polarization (91) and plays a neuroprotective and anti-inflammatory role. After the NSCs transduced by circRNA-HIPK2-siRNA were injected into the lateral ventricle of MCAO mice, NSCs were observed to migrate and differentiate into the ischemic hemisphere, representing a neuroprotective effect which not only increased the plasticity of neurons after ischemia but also significantly reduced functional defects (92). The application of extracellular vesicles is helpful to deliver ncRNA-related preparations into the brain, which is one of the research hotspots of targeted therapy. In animal experiments, intraventricular injection of bone marrow mesenchymal cell exosomes overexpressing miR-223-3p can reduce the size of the infarct area caused by MCAO (93). The exosomes loaded with miR-210 could target the necrotic area after intravenous injection. After 2 weeks, the expressions of VEGF and CD34 were significantly upregulated, and the survival rate of animals was also improved. Yang et al. used engineered rabies virus glycoprotein-circSCMH1-extracellular vesicles to transport circRNA-SCMH1 into the brain (87) and found that the functional recovery after IS was promoted significantly. After transnasal administration of RVG29-modified microRNA-loaded nanoparticles (94), it was seen that cerebral ischemia-reperfusion injury was reduced. RVG29 is derived from the rabies virus glycoprotein and has shown efficient brain-targeted drug delivery and a good safety profile during its use. Transnasal delivery of targeted drugs is expected to be an effective treatment for neurological disorders.

Some drugs have also been found to cause changes in the expression of ncRNAs during the application with potential therapeutic effect on IS. Dexmedetomidine (DEX) is a good anesthetic sedative. At present, some studies have found that the application of DEX seems to be beneficial to the prognosis of IS (95). After DEX application, the low expression of miR-381 was reversed. Accordingly, the expression of miR-381 downstream molecules IRF4 and IL-9 decreased, showing smaller infarct foci. Sevoflurane (SEVO) is considered a neuroprotective agent for cerebral ischemia-reperfusion injury (96). After treatment with SEVO, the expression of miR-181a decreased in MCAO rats, increasing the expression of XIAP in cortical neurons and decreasing the release of Lactate Dehydrogenase (LDH), thereby showing better cell survival. Cao et al. showed that the expression of lncRNA-MALAT1 and downstream molecule HMGB1 decreased after preventive treatment with berberine (BBR) in MCAO mice (36), suggesting that BBR may be involved in the anti-inflammatory effect after inducing IS. Notably, these drugs are not directly targeting agents of ncRNAs. Relevant studies can only hint that the drug effect is related to the ncRNA regulatory network, but this may provide information for studying drug mechanisms and the development of targeted drugs.

## Challenges and Perspectives

Even from the incomplete understanding of the role of ncRNAs in IS, ncRNAs are widely involved in the gene regulatory network, particularly in regulating the critical process of disease onset and development. However, most studies start with a single molecule to explore its upstream or downstream molecules to construct a single pathway that is scattered. Future studies should focus more on the relationship between ncRNAs to form a complete ncRNA regulatory network. Research shows that ncRNA after IS can be detected in serum and possess disease specificity, conveying the potential to be used as early biomarkers of IS. However, whether ncRNAs are abnormally expressed before the onset of IS remains to be researched to help therapists identify and intervene early to prevent serious consequences. A comprehensive understanding of the interaction between ncRNA nodes, such as feedback loop and antagonism, is vital for applying the research results to clinical treatment. This is because single-dose targeted therapy can sometimes prove to be insufficient and combination therapy requires consideration of the interactions between drug pathways of action (97). Hence, the multi-temporal changes of molecular and cellular state changes after IS, targeting, and safety of ncRNAs agents are issues that need to be addressed by researchers.

More work is still demanded to describe ncRNA regulatory networks to help understand the onset of IS and provide information for selecting better therapeutic targets. The application of bioinformatics has dramatically promoted the interpretation of the interaction between ncRNA nodes and other signal pathway members, presenting great benefits to the study of complex ncRNA regulatory networks. With the help of computers, unlocking this complex network may just be around the corner. It is inevitable for ncRNA to become a hotspot with good prospects in basic and clinical research because of its significant role. The identification and development of the ncRNA regulatory network may change our understanding and treatment of diseases.

## Author Contributions

ZC and SL contributed to the investigation and wrote the original draft of the manuscript. TY and JD contributed to the revised version of our manuscript. XL contributed to the methodology. JJ contributed to the conceptualization. All authors read and approved the final manuscript.

## Funding

This work was supported by the Cuiying Scientific and Technological Innovation Program of Lanzhou University Second Hospital (CY2018-QN17), Fundamental Research Funds for the Central Universities (lzujbky-2019-sp04), Gansu Province Youth Science and Technology Fund Project (20JR5RA318), the National Natural Science Foundation of China Regional Foundation Project (82060400), and the Cuiying Scientific Training Program for Undergraduates of Lanzhou University Second Hospital (CYXZ2021-01).

## Conflict of Interest

The authors declare that the research was conducted in the absence of any commercial or financial relationships that could be construed as a potential conflict of interest.

## Publisher's Note

All claims expressed in this article are solely those of the authors and do not necessarily represent those of their affiliated organizations, or those of the publisher, the editors and the reviewers. Any product that may be evaluated in this article, or claim that may be made by its manufacturer, is not guaranteed or endorsed by the publisher.
